# Potential Use of ChatGPT in Responding to Patient Questions and Creating Patient Resources

**DOI:** 10.2196/48451

**Published:** 2024-03-06

**Authors:** Kelly Reynolds, Trilokraj Tejasvi

**Affiliations:** 1 Department of Dermatology University of Michigan Ann Arbor, MI United States

**Keywords:** artificial intelligence, AI, ChatGPT, patient resources, patient handouts, natural language processing software, language model, language models, natural language processing, chatbot, chatbots, conversational agent, conversational agents, patient education, educational resource, educational

## Abstract

ChatGPT (OpenAI) is an artificial intelligence–based free natural language processing model that generates complex responses to user-generated prompts. The advent of this tool comes at a time when physician burnout is at an all-time high, which is attributed at least in part to time spent outside of the patient encounter within the electronic medical record (documenting the encounter, responding to patient messages, etc). Although ChatGPT is not specifically designed to provide medical information, it can generate preliminary responses to patients’ questions about their medical conditions and can precipitately create educational patient resources, which do inevitably require rigorous editing and fact-checking on the part of the health care provider to ensure accuracy. In this way, this assistive technology has the potential to not only enhance a physician’s efficiency and work-life balance but also enrich the patient-physician relationship and ultimately improve patient outcomes.

## Introduction

ChatGPT (OpenAI) is an artificial intelligence (AI)–based natural language processing model that leverages data via complex deep learning algorithms to generate human-like text responses to user-generated prompts [1,2]. This tool is able to quickly, and often remarkably and accurately, generate responses to complex prompts across an infinite array of topics [1,2]. Since the rollout of ChatGPT in November 2022, it has garnered a significant amount of attention for its ability to create remarkably astute prompts for complex inquiries, making it an incredible tool not only for personal use but also for professional and commercial use [1-3].

It is difficult to overstate how the application of ChatGPT and other AI assistive technologies will revolutionize so many aspects of our day-to-day lives. Specifically for health care providers, it seems that there are myriad ways in which this writing-assistant technology has the potential to not only enhance a physician’s efficiency and work-life balance but also enrich the patient-physician relationship and ultimately improve patient outcomes. The advent of this assistive technology has come at a dire time for health care providers, as burnout is at an all-time high [4]. A study funded by the Agency for Healthcare Research and Quality found that the electronic medical record (EMR) is a key player in promoting stress and physician burnout, specifically time spent in the EMR outside of the patient encounter. The Agency for Healthcare Research and Quality [5] has proposed a variety of interventions to mitigate this issue, including offloading of physicians’ workload as well as implementation of simplified, standardized, and automated workflow operations within the EMR, and preliminary applications of AI in these processes have shown promise [6]. Specifically, we propose that ChatGPT may be a promising tool to help reduce time spent outside of the patient encounter for dermatologists and other outpatient health care providers by helping to generate first drafts of written information for patients—for example, instructions for patients and responses to questions in the “patient portal”—which seems to be a relatively underexplored application of this technology.

Although there is a buzz of excitement regarding the application of ChatGPT and other algorithmic or AI technologies in science and medicine [2,7], this excitement is balanced by important concerns about the limitations of this technology or fears about these algorithmic technologies outperforming or replacing health care providers. Importantly, although algorithms have their rightful place in the practice of medicine, the use of algorithms does not substitute for clinical judgment and does not capture the nuances of individualized medicine. This speaks to the importance of the patient-physician relationship, which is based on subtleties in human interactions that AI technologies cannot capture [6]. There are also important ongoing conversations regarding the ethical, privacy, and regulatory concerns about the use of AI technology in health care, although an in-depth conversation on these apprehensions is beyond the scope of this paper [6,7].

An obvious but important caveat is that ChatGPT in its current form is not designed to provide medical information. If, as a patient, you ask a medical question, ChatGPT implements a stock phrase: “As an AI language model, I am not authorized to provide personalized medical advice or recommendations. It is important to consult with a qualified healthcare professional...” [1]. This new technology is still “learning” and may occasionally “hallucinate,” that is, make up false information. Additionally, in generating its responses, ChatGPT may reference poor-quality studies or those with disproven conclusions and may not account for recently published research. This potentially dangerous limitation has been a focus of many critics of this technology, as ChatGPT-generated false medical information could be misleading, and broader dissemination thereof has the potential to create widespread confusion and potentially foster distrust of medical providers [6]. While solely relying on AI-generated health information without human oversight does indeed have dangerous potential, we propose that if ChatGPT-generated responses are edited and fact-checked by a human health care provider, the ChatGPT-based synthesis of information can be an incredibly useful tool to generate a first draft for patient-based resources.

For example, a patient sends a message to his dermatologist inquiring about whether he should stop his bexarotene for treatment of his cutaneous T-cell lymphoma after having been diagnosed with COVID-19. Inputting this question into ChatGPT generates an accurate response; of course, the caveat is that the chatbot is “not authorized to give medical advice.” Adding more specificity to the prompt specifying that the response should be at a “fifth-grade reading level” or should “cite scientific papers” creates an even better response (Textbox 1 [1]).

After reviewing and making edits to the ChatGPT output, this thorough response may be as good or superior to the response that many dermatologists would have been able to generate de novo.

This same approach can also be applied to drafting written resources for patients. For example, prompting ChatGPT to “create a resource for a patient at a fifth-grade reading level regarding the special considerations of topical steroid application in patients with autism” yields a response (Textbox 2 [1]) in seconds, which would have otherwise required a fair amount of effort to create from scratch.

**ChatGPT’s response to a patient question regarding the discontinuation of bexarotene in the setting of active COVID-19 infection.**

“If you have been diagnosed with COVID-19, it’s important to talk to your doctor before stopping any medication. There is currently no scientific evidence suggesting that bexarotene should be stopped if you have COVID-19.

A study published in the Pharmacological Research journal found that bexarotene may have antiviral effects against COVID-19 [8]. However, more research is needed.

In conclusion, if you have been diagnosed with COVID-19 and are taking bexarotene, it’s important to talk to your doctor before stopping the medication. There is currently no scientific evidence suggesting that bexarotene should be stopped if you have COVID-19, and it may even have some potential benefits.”

**ChatGPT’s response to a patient question regarding topical steroid application considerations in patients on the autism spectrum.**

“Title: Understanding Topical Steroid Application in Patients with Autism

Introduction: If you or a loved one has autism and needs to use topical steroids, there are a few things you should know. Topical steroids are medicines that are applied to the skin to reduce inflammation and itching. This resource will explain the special considerations that people with autism should keep in mind when using topical steroids.

What are Topical Steroids? Topical steroids are medicines that are applied to the skin to reduce inflammation and itching. They are often used to treat skin conditions like eczema, psoriasis, and allergic reactions. Topical steroids come in many forms, including creams, ointments, and lotions.

Special Considerations for People with Autism: If you or your loved one has autism and needs to use topical steroids, here are some things to keep in mind:

Communication: People with autism may have difficulty communicating their symptoms or the effects of the medication. It is important to communicate any changes in symptoms or side effects to your doctor.

Sensory Issues: People with autism may be sensitive to touch, texture, or smell. They may have difficulty with the feel or smell of the medication. Talk to your doctor about any sensory issues you or your loved one may have.

Routine: People with autism often thrive on routine. Applying medication at the same time each day can help establish a routine and make the medication easier to manage.

Education: Educate yourself and your loved one about the medication. Explain why the medication is necessary and what it is intended to do. Use simple language and visuals if needed.

Conclusion: Topical steroids can be a helpful treatment for skin conditions, but special considerations need to be taken for people with autism. If you or your loved one has autism and needs to use topical steroids, communicate any changes in symptoms or side effects to your doctor, address any sensory issues, establish a routine, and educate yourself and your loved one about the medication. Talk to your doctor if you have any questions or concerns.”

## Conclusions

In conclusion, ChatGPT is a tool that may show promise in assisting dermatologists and other outpatient health care providers in generating information for patients to address their questions and concerns, which may help patients better understand and manage their condition and ultimately may help to promote treatment compliance and enhance the patient-physician relationship. The use of ChatGPT has important implications as it pertains to reducing physician burnout by assisting with and automating tasks outside of the patient encounter. Although there are important limitations to this technology, by ensuring the prompt is specific and using this output as a “first draft” and editing for accuracy, this technology can be used to generate exceptionally high-quality patient resources. Importantly, ensuring the accuracy and appropriateness of the medical information generated by ChatGPT requires rigorous evaluation and validation against trusted sources as well as adherence to current regulatory guidelines. There are seemingly endless ways in which natural language processing tools such as ChatGPT may be used to streamline health care providers’ workflow, thereby reducing burnout. However, more research is needed regarding patients’ perceptions of chatbot-generated resources as well as the potential implications of AI on the patient-physician relationship.

## Abbreviations

AI: artificial intelligence
EMR: electronic medical record
